# Supplementary material for: Content-rich biological network constructed by mining PubMed abstracts
Source: BMC Bioinformatics. 2004 Oct 8;5:147. doi: 10.1186/1471-2105-5-147 (PMC528731; doi:10.1186/1471-2105-5-147)
Supplement: Additional File 2 — The original results of the above study (non-essential files are deleted to keep the file size under the limit set by BMC bioinformatics). [file 1471-2105-5-147-S2.bz2 › chilibotAdditionalFile2/dip20/345ID9261146E4421/html/IL9_IL9R.html]

 


 **IL9** and **IL9R** 
  
Found 39 abstracts in PubMed, retrieved 20.  
 

 What does Google say? 
 PDF only 
| .edu only 

---

**Interactive relationship** (e.g. stimulation, inhibition, etc)

**Inhibitory relationship**- Human growth factor dependent cell line TF1, which lacks interleukin IL 9  [ **IL9** ]  receptors R and does not grow in IL 9  [ **IL9** ] , was transduced with a retroviral vector containing human IL 9R  [ **IL9R** ]  cDNA and a selection marker.  Ref: 10738974 J Hematother Stem Cell Res, 2000
**Stimulatory relationship**- An increased number and size of burst forming units erythroid BFU E like colonies were detected from IL 9R  [ **IL9R** ]  transduced TF1 cells, compared with mock transduced cells, in response to erythropoietin EPO and IL 9  [ **IL9** ] .  Ref: 10738974 J Hematother Stem Cell Res, 2000
**Neutral relationship**- Interleukin 9  [ **IL9** ]  IL 9  [ **IL9** ]  exerts its pleiotropic effects through the IL 9  [ **IL9** ]  receptor IL 9R  [ **IL9R** ]  complex, which consists of the IL 9R  [ **IL9R** ]  alpha chain, which determines the cytokine specificity, and the IL 2 receptor gamma chain.  Ref: 10642536 Biochem J, 2000
- Interleukin 9  [ **IL9** ]  IL 9  [ **IL9** ]  exerts its pleiotropic effects through the IL 9  [ **IL9** ]  receptor IL 9R  [ **IL9R** ]  complex that consists of the ligand specific IL 9R  [ **IL9R** ]  alpha chain, and the IL 2R gamma chain.  Ref: 10486269 Biochem Biophys Res Commun, 1999

**Non-interactive relationship** (e.g. studied together, co-existance, homology, etc.)

- In this study, we evaluated expression of the IL 9R  [ **IL9R** ]  and the effect of IL 9  [ **IL9** ]  on human PMNs.  Ref: 11160343 J Immunol, 2001
- Reverse transcriptase polymerase chain reaction of BEAS 2B, HBE4 E6 E7, and primary cells from two subjects revealed messenger RNA for IL 9  [ **IL9** ]  receptor IL 9R  [ **IL9R** ]  alpha but not in BEAS 2B cells.  Ref: 11588013 Am J Respir Cell Mol Biol, 2001
- OBJECTIVE To investigate the contribution of IL 9  [ **IL9** ]  to the pathogenesis of asthma, we examined the expression of IL 9  [ **IL9** ]  and its receptor IL 9R  [ **IL9R** ]  in bronchial tissue from subjects with atopic asthma n = 10, chronic bronchitis n = 11, and sarcoidosis n = 9 and from atopic n = 7 and nonatopic n = 10 healthy control subjects.  Ref: 10629460 J Allergy Clin Immunol, 2000
- The IL 9  [ **IL9** ]  receptor IL 9R  [ **IL9R** ]  belongs to the haemopoietic receptor superfamily and its signal transduction involves mainly the Janus kinase signal transducer and activator of transcription JAK STAT pathway.  Ref: 11115404 Biochem J, 2001
- TF1 cells stably expressing IL 9R  [ **IL9R** ]  and responding to IL 9  [ **IL9** ]  can serve as a cell line model to study the intracellular signals mediating IL 9  [ **IL9** ]  induced erythroid cell proliferation and differentiation.  Ref: 10738974 J Hematother Stem Cell Res, 2000
- Since our genetic analysis of the 5q cytokine cluster did not support a genetic role for the  **IL9**  gene, we became interested in the  **IL9**  receptor gene  **IL9R**  in the pseudoautosomal region.  Ref: 11039580 Eur J Hum Genet, 2000
- Analysis of IL 9R  [ **IL9R** ]  alpha subunit expression on 18 T cell clones revealed that IL 9  [ **IL9** ]  responsiveness was directly proportional to the expression of the high affinity receptor.  Ref: 10888117 J Interferon Cytokine Res, 2000
- CONCLUSIONSIncreased expression of IL 9  [ **IL9** ]  and IL 9R  [ **IL9R** ] , as well as upregulation of hCLCA1, in mucus overproducing epithelium of patients with cystic fibrosis supports the hypothesis that IL 9  [ **IL9** ]  contributes to mucus overproduction in cystic fibrosis.  Ref: 12782818 Laryngoscope, 2003
- IL 9  [ **IL9** ]  and IL 9R  [ **IL9R** ]  positive cells were identified with immunocytochemistry, and HCLCA1 expression was detected by means of in situ hybridization with cRNA probes.  Ref: 11842292 J Allergy Clin Immunol, 2002
- METHODS Bronchial biopsy specimens were examined for the presence of IL 9  [ **IL9** ]  and IL 9R  [ **IL9R** ]  protein and messenger RNA mRNA by immunocytochemistry and in situ hybridization, respectively.  Ref: 10629460 J Allergy Clin Immunol, 2000
- IL 9  [ **IL9** ]  responsiveness was also dependent on long term culturing because neither freshly isolated nor 3 day phytohemagglutinin PHA stimulated peripheral blood lymphocytes PBL expressed IL 9R  [ **IL9R** ]  alpha.  Ref: 10888117 J Interferon Cytokine Res, 2000
- To examine the interaction between IL 9  [ **IL9** ]  and eosinophils, we evaluated mature peripheral blood eosinophils for their expression of the specific alpha subunit of the IL 9  [ **IL9** ]  receptor IL 9R  [ **IL9R** ]  alpha.  Ref: 10979962 Blood, 2000
- We sought to examine the expression of IL 9  [ **IL9** ] , interleukin 9  [ **IL9** ]  receptor IL 9R  [ **IL9R** ] , and hCLCA1 in the upper airway of patients with cystic fibrosis in comparison to healthy control subjects and to demonstrate the relationship of IL 9  [ **IL9** ] , IL 9R  [ **IL9R** ] , and hCLCA1 expression with mucus production.STUDY DESIGNProspective design.METHODSBiopsy samples from nasal polyps of four patients with cystic fibrosis, nasal mucosa of six patients with cystic fibrosis, sinus mucosa of eight patients with cystic fibrosis, and nasal mucosa of six healthy control subjects were stained with periodic acid Schiff PAS to identify mucus glycoconjugates.  Ref: 12782818 Laryngoscope, 2003
- IL 9  [ **IL9** ] , IL 9R  [ **IL9R** ] , and hCLCA1 expression was determined by immunocytochemical study.RESULTSWe demonstrated significant increases in IL 9  [ **IL9** ] , IL 9R  [ **IL9R** ] , and hCLCA1 immunoreactivity in the mucosa of patients with cystic fibrosis compared with that found in control subjects P < ;.  Ref: 12782818 Laryngoscope, 2003
- OBJECTIVE We sought to examine the expression of HCLCA1 in bronchial biopsy specimens of asthmatic subjects compared with those of control subjects and to demonstrate its relationship with IL 9  [ **IL9** ] , IL 9  [ **IL9** ]  receptor IL 9R  [ **IL9R** ] , and markers of mucus production.  Ref: 11842292 J Allergy Clin Immunol, 2002
